# Is There a Relationship between Ovarian Epithelial Dysplasia and Infertility?

**DOI:** 10.1155/2012/429085

**Published:** 2012-02-09

**Authors:** Gautier Chene, Frédérique Penault-Llorca, Anne Tardieu, Anne Cayre, Nicole Lagarde, Patricia Jaffeux, Bruno Aublet-Cuvelier, Pierre Dechelotte, Bertrand Felloni, Jean-Luc Pouly, Jacques Dauplat

**Affiliations:** ^1^Department of Histopathology, Centre Jean Perrin, Clermont-Ferrand 63000, France; ^2^Department of Surgery, Centre Jean Perrin, Clermont-Ferrand 63000, France; ^3^Department of Obstetric, Gynecology and Reproductive Medicine, CHU St. Etienne, Saint-Etienne 42000, France; ^4^Department of Medical Information, CHU Clermont-Ferrand, Clermont-Ferrand 63000, France; ^5^Department of Histopathology, CHU, Clermont-Ferrand 63000, France; ^6^Department of Obstetric, Gynecology & Reproductive Medicine, CHU Clermont-Ferrand, Clermont-Ferrand 63000, France

## Abstract

*Aim.* Ovarian epithelial dysplasia was initially described in material from prophylactic oophorectomies performed in patients at genetic risk of ovarian cancer. Similar histopathological abnormalities have been revealed after ovulation stimulation. Since infertility is also a risk factor for ovarian neoplasia, the aim of this study was to study the relationship between infertility and ovarian dysplasia. *Methods.* We blindly reviewed 127 histopathological slides of adnexectomies or ovarian cystectomies according to three groups—an exposed group to ovulation induction (*n* = 30), an infertile group without stimulation (*n* = 35), and a spontaneously fertile control group (*n* = 62)—in order to design an eleven histopathological criteria scoring system. *Results.* The ovarian dysplasia score was significantly higher in exposed group whereas dysplasia score was low in infertile and control groups (resp., 8.21 in exposed group, 3.69 for infertile patients, and 3.62 for the controls). In the subgroup with refractory infertility there was a trend towards a more severe dysplasia score (8.53 in ovulation induction group and 5.1 in infertile group). *Conclusion.* These results raise questions as to the responsibility of drugs used to induce ovulation and/or infertility itself in the genesis of ovarian epithelial dysplasia.

## 1. Introduction

Histopathological study of material from prophylactic oophorectomies performed for a genetic predisposition of ovarian cancer revealed cytological and architectural abnormalities considered to be precancerous manifestations, and termed “dysplasia” by analogy with the pre-invasive lesions described for the genital tract (vulva, vagina, cervix, endometrium) [1]. Several studies have found similar ovarian dysplasia lesions after stimulation of ovulation in infertile patients, without any indication of their long-term evolution [2, 3]. However, the relationship between ovulation induction and ovarian dysplasia is not obvious because infertility in itself represents a confounding factor [3]. The question is whether the lesions are somehow related to the infertility or are due to the ovulation stimulation.

The aim of this study is to determine the relationship between infertility and ovarian epithelial dysplasia.

## 2. Methods

### 2.1. Patients

Using a database covering 1.400 adnexectomies and/or ovarian cystectomies carried out between January 1995 and December 2000, we selected three groups.

**Group A: An Exposed Group**

Who had adnexectomies and/or ovarian cystectomies after in vitro fertilization using ovulation induction several years later and whose ovaries were reported as normal on routine histological examination. We felt it would be interesting to study more particularly those cases in which there will be failure of stimulation. We called this subgroup “refractory infertility.”

**Group B: An Infertile Group**

Without ovulation induction who had adnexectomies and/or ovarian cystectomies prior to any assisted reproductive treatment (ART) technique and whose ovaries were reported as normal on routine histological examination. They did not receive ovulation induction before the surgery. We felt it would be interesting to study more particularly those cases of infertility in which there will be failure of stimulation. We called this sub-group “refractory infertility.”

**Group C: Control Group**

We selected a spontaneously fertile population, with no personal nor family history of gynaecologic neoplasia (breast, ovary, endometrium), who underwent adnexectomy and/or cystectomy for which the histopathological examination concluded that the ovaries showed no sign of cancerous or borderline pathology.

### 2.2. Histopathological Criteria

**Figure 1:**
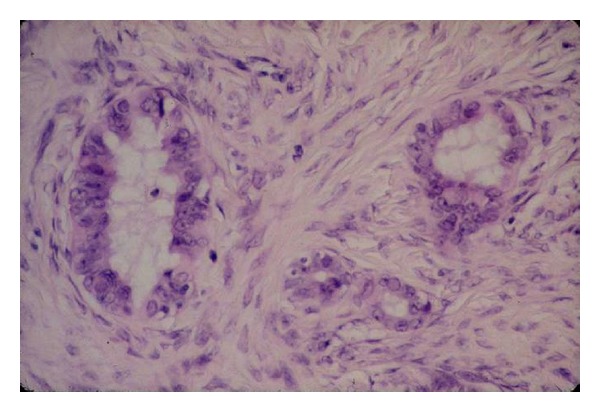
Nuclear abnormalities in ovarian epithelial dysplasia (HES, ×40), from Dr. L. Deligdisch collection.

Our definition of ovarian atypia was based on previous studies of ovarian dysplasia, that is, dysplasia described in ovaries from patients with a genetic risk (prophylactic oophorectomy for BRCA1/2 mutation) [4–6], in areas that appeared to be “healthy” adjacent to an ovarian cancer [7, 8], in the apparently healthy contralateral ovary in case of unilateral ovarian cancer [9, 10], and in stimulated ovaries [2, 3]. This scoring system (eleven histopathological criteria) was designed in our previous study about the relationship between ovarian dysplasia and ovulation induction [3]:

epithelial pseudostratification,
epithelial proliferation,
surface papillomatosis,
irregular nuclear chromatin pattern (Figure 1),
irregular nuclear contour,
cellular pleiomorphism,
increase in nuclear size
inclusion cysts,
deep epithelial invaginations,
psammoma,
stromal hyperplasia.

In each case, the least normal area was given a score between 0 and 2 (0: normal, 1: moderately abnormal, 2: severely abnormal), whether located on the surface or in an inclusion cyst.

An overall dysplasia score was then obtained for each patient by simply adding the scores for each of the 11 items (total range: 0 to 22).

Morphological studies were processed on 3 micron paraffin sections stained with standard haematoxylin phloxin safran (HPS). The number of sections available for review for each case ranged from 8 to 11 in both study groups.

The histopathology slides were all reexamined blinded by two pathologists who were expert in oncogynaecology. When several slides were available, the one with the highest dysplasia score was retained. Concerning the cystectomies, the slides were re-examined on the one hand to confirm the histopathological diagnosis and on the other to look for associated ovarian tissue in order to establish the dysplasia score. If there was no ovarian tissue the file was excluded.

In the event of obvious differences between the scores established by each pathologist, a further examination was carried out to reach a consensus.

### 2.3. Statistical Analysis

Our main measurement was the mean dysplasia score. Student's *t*-test was used to compare the dysplasia score means of both groups. 

## 3. Results

All the included patients are eligible.

30 exposed patients (group A), 35 infertile patients (group B), and 62 fertile controls (group B) were included in the study. There were 18 “refractory infertility”patients in group A and 21 “refractory infertility” patients in group B. The characteristics and the indications of surgery of the three groups are given in Table 1.

Histopathological features of excised material from group A, B, and C were mainly benign cysts (resp., 30 cysts, 32 cysts, and 40 cysts) without cancer or borderline tumor. Histopathological analysis is given in Table 2.

Infertility was female in 70% of cases in group A and 71% of cases in group B, with the following distribution: in group A, ovarian dysovulation 10%, tubal pathology 40%, endometriosis 50% and in group B, ovarian dysovulation 4%, tubal pathology 36%, and endometriosis 60%.

The cytological and architectural abnormalities of the ovarian epithelium described by our score were always assessed in the ovarian tissue. The histopathological abnormalities in both groups are described in Table 3. Histopathological anomalies were always present in group A whereas they were rare in group B.

Based on this data, a mean dysplasia score was determined for both groups: 8.21 for group A, 3.69 for the infertile patients, and 3.62 for the controls. The difference was statistically different between group A and C (**P** < 0.0001). However, there was no significant difference between group B and C (**P** = 0.92), nor were any statistically significant differences found according to the aetiology of infertility.

In the “refractory sterility” group, the dysplasia score was higher in group A than in group B: 8.53 for group A and 5.1 for group B, **P** = 0.02.

An estimate of the study's power is 0.99.

## 4. Discussion

Ovarian dysplasia was initially described in ovaries with a genetic risk of cancer [1, 4, 5]. By analogy with preinvasive cervical lesions, the generic term “dysplasia” was proposed. The fact that these ovaries could evolve towards malignancy if prophylactic ovariectomy did not take place led to the idea that ovarian epithelial dysplasia was the missing link prior to neoplasia.

More recently similar ovarian lesions described as dysplasia were detected in ovaries stimulated during IVF treatment. Nieto et al. [2] were the first to find significant abnormalities in stimulated ovaries compared to a control population. One of our previous studies confirmed these results (mean dysplasia score 7.64) and also showed that a time effect and a dose effect were probable [3]: histopathologic abnormalities (cf photo) would become more severe and greater in number with an increasing number of stimulation cycles (>3) and after a sufficient lapse of time (over seven years). However it is impossible to predict how they would evolve: the dysplastic profile of stimulated ovaries and ovaries with genetic risk is not the same, which would tend to indicate a different evolution at long term [11, 12]. Animal experiments gave some interesting conclusions. Ovulation in rats has resulted in increased Ki67 expression and dysplastic abnormalities in the ovarian epithelium [13]. Çelik et al. [14] found also a relationship between the number of ovulation-inducted cycles and the severity of ovarian dysplasia: when comparing the rate of ovarian dysplasia in three groups of rats subjected to one, three, and six gonadotrophin cycles, there was a significant trend towards more severe dysplasia as the number of inducted ovulation cycles increased. Ozcan et al. [15] have examined the effects of ovulation induction agents on ovarian epithelium after 6 and 12 cycles: ovarian dysplasia (more severe after 12 cycles) was found to be significant in the ovaries of rats that were given clomiphene citrate, recombinant FSH, and human menopausal gonadotrophin. However no malignant ovarian lesion was found in these three animal studies.

Patients undergoing ovarian stimulation could be at increased risk of ovarian tumors (8.21 versus 3.62): few studies have discussed the possible relationship between exogenous hormones and the risk of developing borderline malignancy of the ovary [11, 12, 16]. Therefore, the discovery of ovarian dysplasia in stimulated ovaries raises the question of the possible responsibility of the treatment used to induce ovulation. So two questions require an answer.

Firstly, is ovarian dysplasia a histopathologic entity, or a simple variant from normal?

Secondly, is infertility or ovulation induction a risk factor for dysplasia, and could it be held responsible for the appearance of dysplastic abnormalities?

(1) One of the major disadvantages of a histopathologic score is that there will be subjectivity when applying it. There is no consensual dysplasia scoring scheme. So we designed an exhaustive scoring system for dysplasia in ovaries at genetic risk and in ovaries in relation with ovulation induction [3, 17, 18]. Although we do not separate cellular changes in inclusion cysts and surface epithelium (ovarian surface epithelial changes are rarer than in inclusion cysts) [19], our histopathological dysplasia score seems to be reproducible (review by several pathologists blinded to clinical data and comparison with control group in order to validate our dysplasia system in one of our previous studies) [20] and consistent with the literature [4–10]. We have proposed a cut-off in one of our latest studies: an ovarian dysplasia score over than 8 (Se: 60%; Sp: 93.3%) [20]. Digitised morphometric analyses based on the degree of stratification and loss of polarity (by measuring the shortest distance between the nucleus and basal membrane, cellular density), and nuclear pleiomorphism (by measurement of the circumference and surface area of the nucleus) [21], or methods of nuclear karyometry (quantitative analysis of nuclear texture) [22, 23] confirm that dysplasia is indeed a distinct histopathologic entity in its own right. Recent immunohistochemistry and molecular studies gave similar results, validating the concept of “ovarian dysplasia” [24, 25].

(2) Human epidemiological studies following up infertile patients have most often demonstrated an increased risk of ovarian tumour (cancerous or borderline), but the results are contradictory: some blame the infertility itself [26, 27] while others lay the blame more on ovulation inducing agents [28, 29].

Our previous studies revealed significant dysplastic lesions in stimulated ovaries [3]. In the present study, there were significant dysplastic lesions in exposed group whereas there was no increase in the dysplasia score in the infertile patients, which is corroborated by the studies of nulliparous patients by Nieto et al. [2].

Should we therefore conclude that treatments to induce ovulation are responsible for the genesis of dysplasia?

Our results show a significant trend towards dysplasia in case of refractory infertility in group A and B. However other cofactors might be involved. Nieto et al. [30] also explored the prevalence of ovarian cancer in patients who were 1st degree relatives of women treated for infertility (due to anovulation) compared with patients who were 1st degree relatives of spontaneously fertile women: the result was a relative risk of 1.45 (95% IC 0.36–10.55) and above all an additional risk in patients who were 1st degree relatives of patients presenting refractory infertility due to dysovulation (14.8, IC 95% 1.36–160). The authors concluded in a probable “genetic link” [30, 31]. A deletion or mutation in a number of genes which regulate cell cycle and cell death in the ovary could affect both fertility (through regulation of follicle pool) and carcinogenesis (by increasing growth stimilus and/or removing growth inhibition): for example, in vitro studies have proved that mice deficient in LATS1 are infertile and develop ovarian tumours [32]. Deletions of Smad1 and Smad5 lead to infertility and ovarian cancer in mice [33].

So our results could corroborate this genetic theory by showing that this sub-group of patients with refractory infertility would be at risk, with the ovulation inducing drugs possibly acting as dysplasia revealers or accelerators.

Caution is needed when interpreting all this data: this is a retrospective observational study with limited numbers of patients. Infertility is a complex and multifactorial pathology in which many confounding factors interfere (age, parity, breastfeeding, dosage level, duration of contraception, etc.) to the point it is difficult to come to any conclusion about risk factor. Although the study's power is very good (0.99), we cannot draw the conclusion that the ovulation stimulation therapy might always cause ovarian dysplasia. However, we can tell that there is sometimes some histopathological abnormalities in ovaries in relationship with ART and infertility. This is a legitimate and very important question that needs more studies not only to describe the dysplastic lesions more precisely but also to look for them in larger series and for their relationships with ovulation inducing drugs.

## 5. Conclusion

This study shows a significant level of abnormalities after ovarian stimulation whereas there is no increase in ovarian epithelial dysplasia in infertile patients compared with fertile patients; we can also note a trend towards a higher incidence of dysplastic changes in “refractory infertility.”

## Figures and Tables

**Table 1:** Characteristics of the study population at the time of surgery.

Variables	Exposed group A	Infertile group B	Control group C
	*N* = 30	*N* = 35	*N* = 62
Age (years)	38,5 (29–50)	30.5 (21–43)	42.1 (32–51)
BMI	23,1	23.4	22.6
Surgical indication			
Metrorrhagia	6	0	20
Pelvic pain	18	8	39
Cyst at ultrasound	30	30	35
Hydrosalpinx	0	3	0
Ovarian biopsies	0	3	0
Nulliparity	5	29	0
Parity	1,1 (1–3)	0 (0-1)	2.7 (1–4)
Use of oral contraception	25, or 83%	28, or 80%	55, or 89%
Duration of exposure to oral contraception (months)	40,1 (4–91)	26.5 (0–134)	58.2 (20–180)

**Table 2:** Histopathological diagnosis on excised tissues.

	Exposed group (group A)	Infertile patients (group B)	Controls (group C)
Pyosalpinx	0	0	5
Hydrosalpinx	0	3*	0
Endometrioma	9	15	12
Serous ovarian cystadenomas	6	3	5
Mucinous ovarian cystadenomas	2	5	6
Ovarian myoma	0	2	5
Follicular ovarian cyst	10	3	9
Haemorrhagic ovarian cyst	3	3	17
Torsion/ischaemia of the adnexa	0	0	3
Ovarian biopsies (ovarian reserve)	0	4*	0

*The 3 patients presenting a hydrosalpinx had several ovarian biopsies.

**Table 3:** Comparison of respective frequencies of the 11 histopathologic abnormalities in our dysplasia scoring system.

	Group A	Group B	Group C	Statistical difference *P*
	*N* = 30	*N* = 35	*N* = 62	
Epithelial pseudostratification	21 (70%)	11 (31.4%)	17 (27.4%)	*P*1 < 0.0001
				*P*2 = 0.98
				*P*3 = 0.002
Epithelial proliferation	16 (53.3%)	8 (22.8%)	23 (37%)	*P*1 = 0.007
				*P*2 = 0.06
				*P*3 < 0.0001
Surface papillomatosis	15 (50%)	7 (20%)	15 (24.1%)	*P*1 = 0.009
				*P*2 = 0.8
				*P*3 < 0.0001
Irregular nuclear chromatine pattern	13 (43.3%)	7 (20%)	18 (29%)	*P*1 = 0.0042 *P*2 = 0.69 *P*3 = 0.001
Irregular nuclear contour	12 (40%)	11 (31.4%)	12 (19.3%)	*P*1 = 0.0012
				*P*2 = 0.059
				*P*3 = 0.08
Cellular pleiomorphism	19 (63.3%)	9 (25.7%)	21 (33.8%)	*P*1 = 0.0078
				*P*2 = 0.32
				*P*3 = 0.0045
Increased size of nucleus	14 (46.6%)	6 (17.1%)	13 (20.9%)	*P*1 = 0.0074
				*P*2 = 0.86
				*P*3 = 0.004
Inclusion cysts	21 (70%)	15 (42.8%)	31 (50%)	*P*1 = 0.032
				*P*2 = 0.22
				*P*3 = 0.004
Psammomas	5 (16.6%)	5 (14.2%)	4 (6.4%)	*P*1 = 0.017
				*P*2 = 0.012
				*P*3 = 0.8
Deep epithelial invaginations	15 (50%)	6 (17.1%)	11 (17.7%)	*P*1 < 0.0001
				*P*2 = 0.99
				*P*3 = 0.002
Stromal hyperplasia	11 (36.6%)	14 (40%)	10 (16.1%)	*P*1 < 0.0001
				*P*2 = 0.0013
				*P*3 = 0.78

*P*1: statistical differences between group A and C. *P*2: statistical differences between group B and C. *P*3: statistical differences between group A and B. Statistical analysis by Student's *t*-test.
